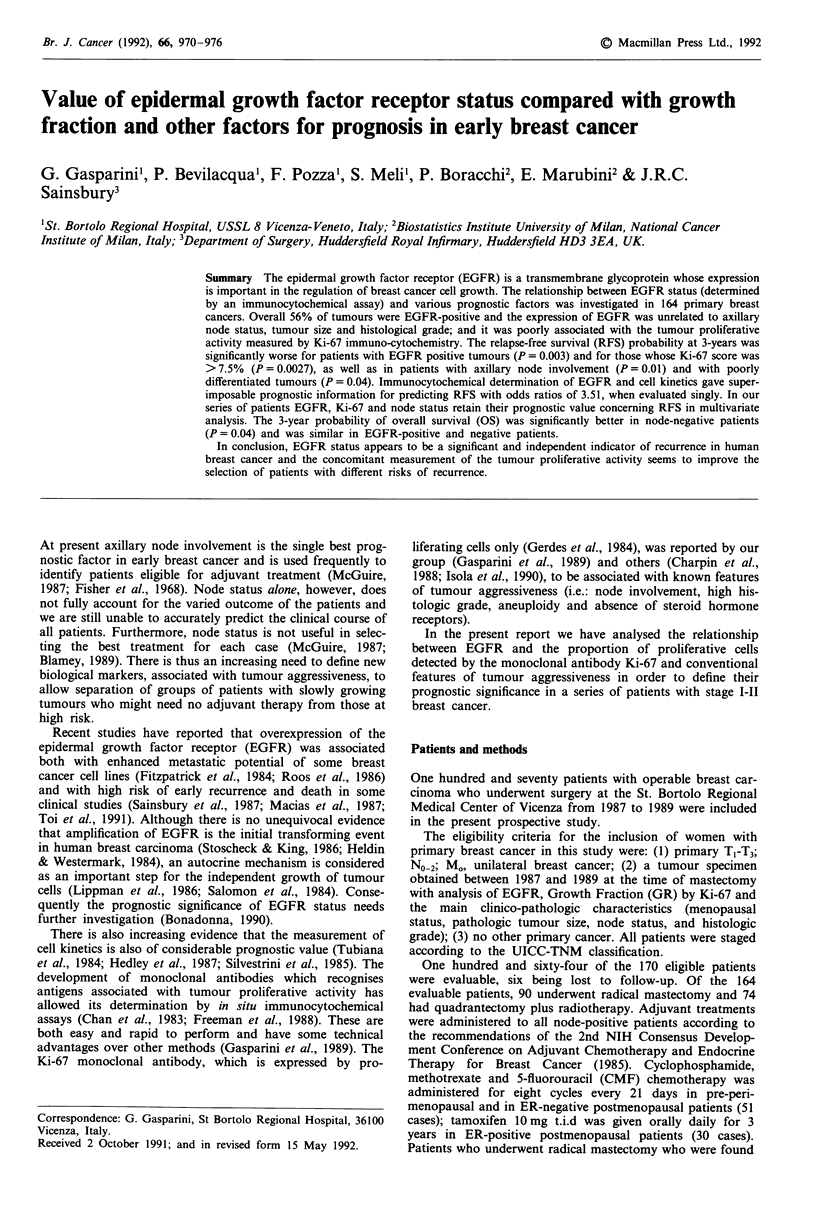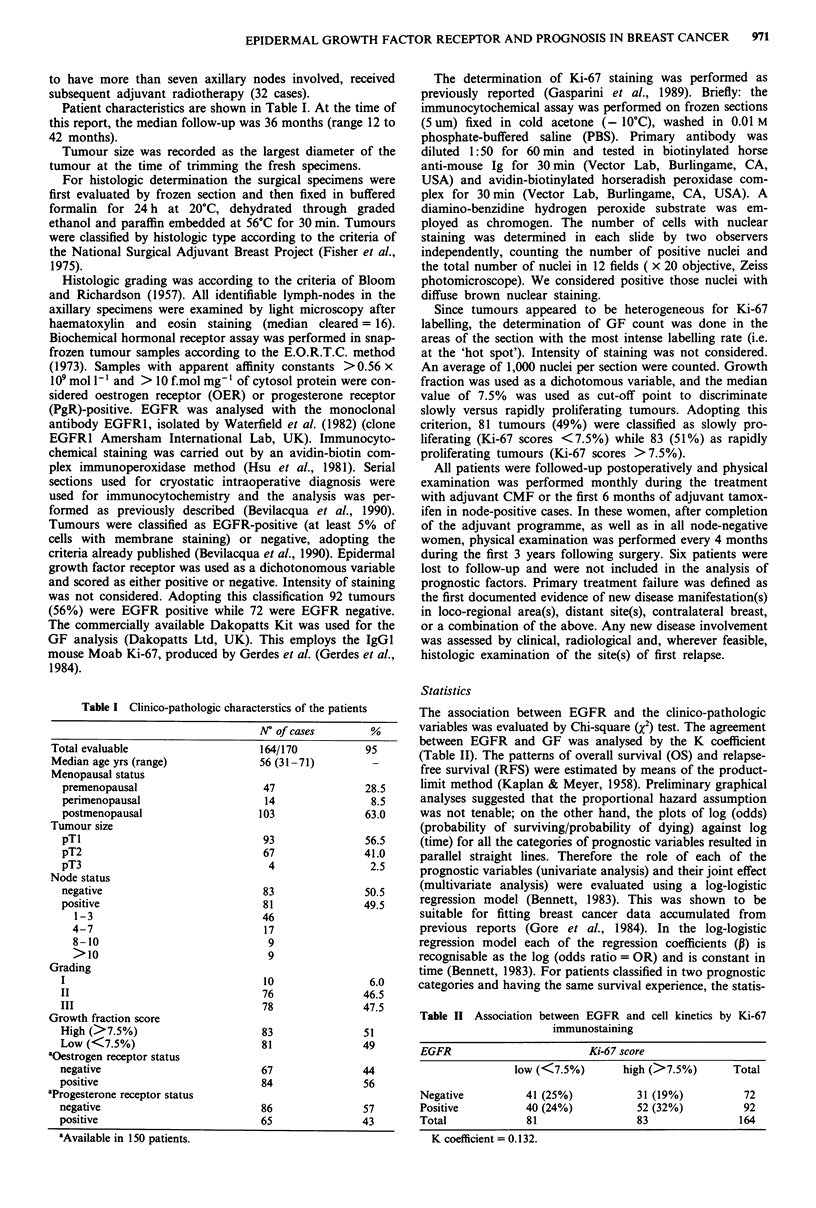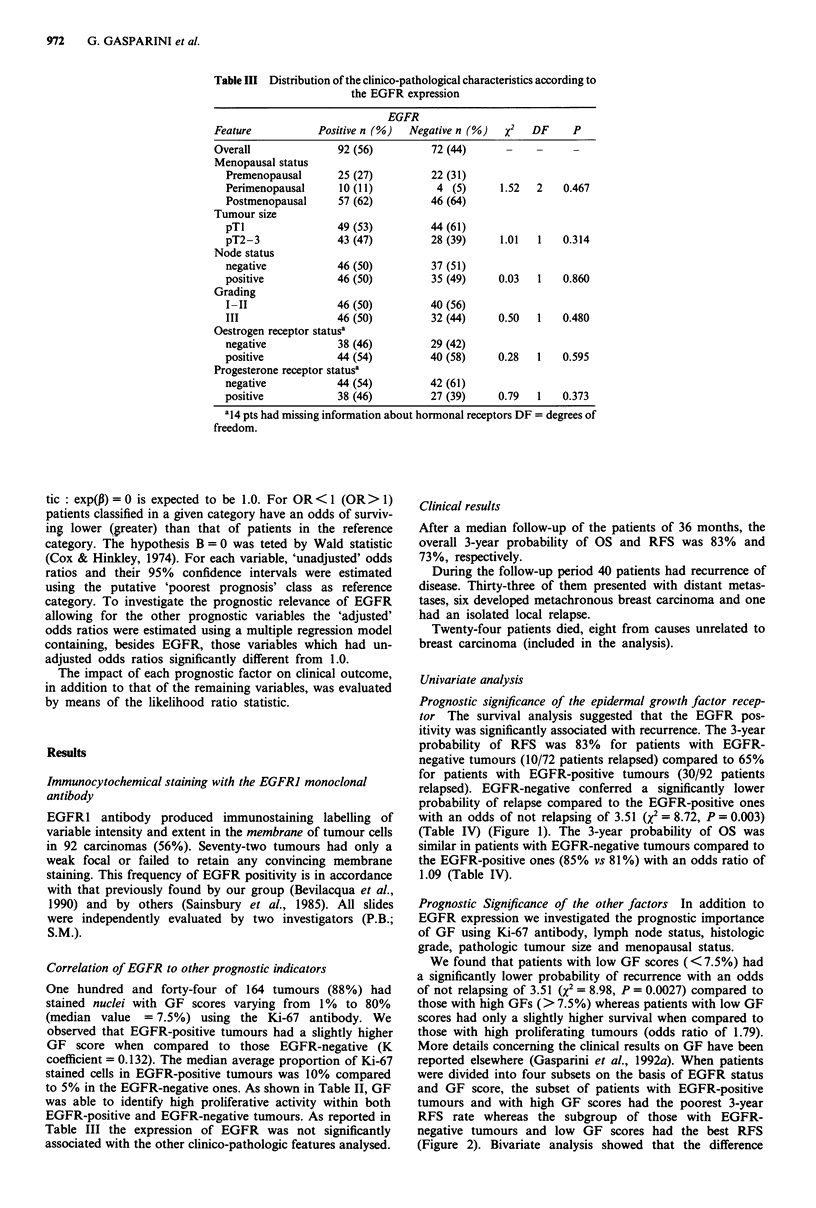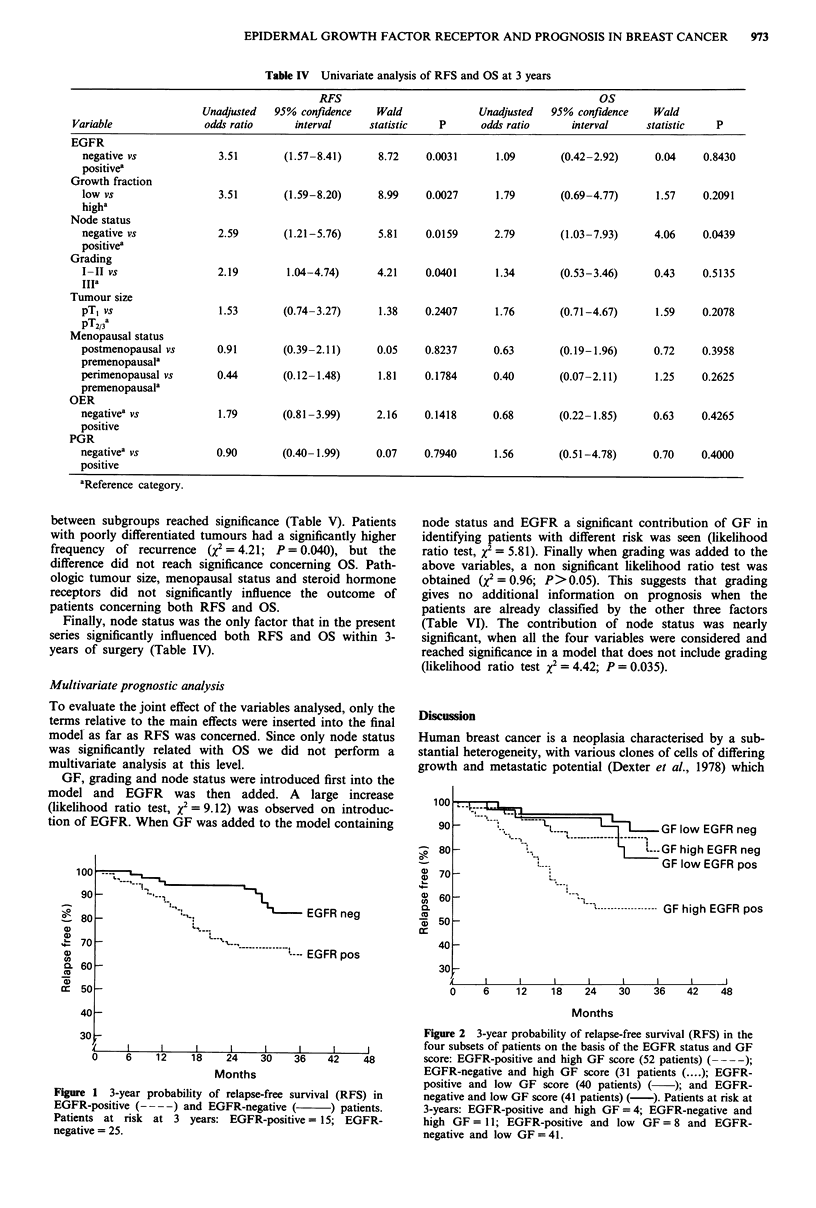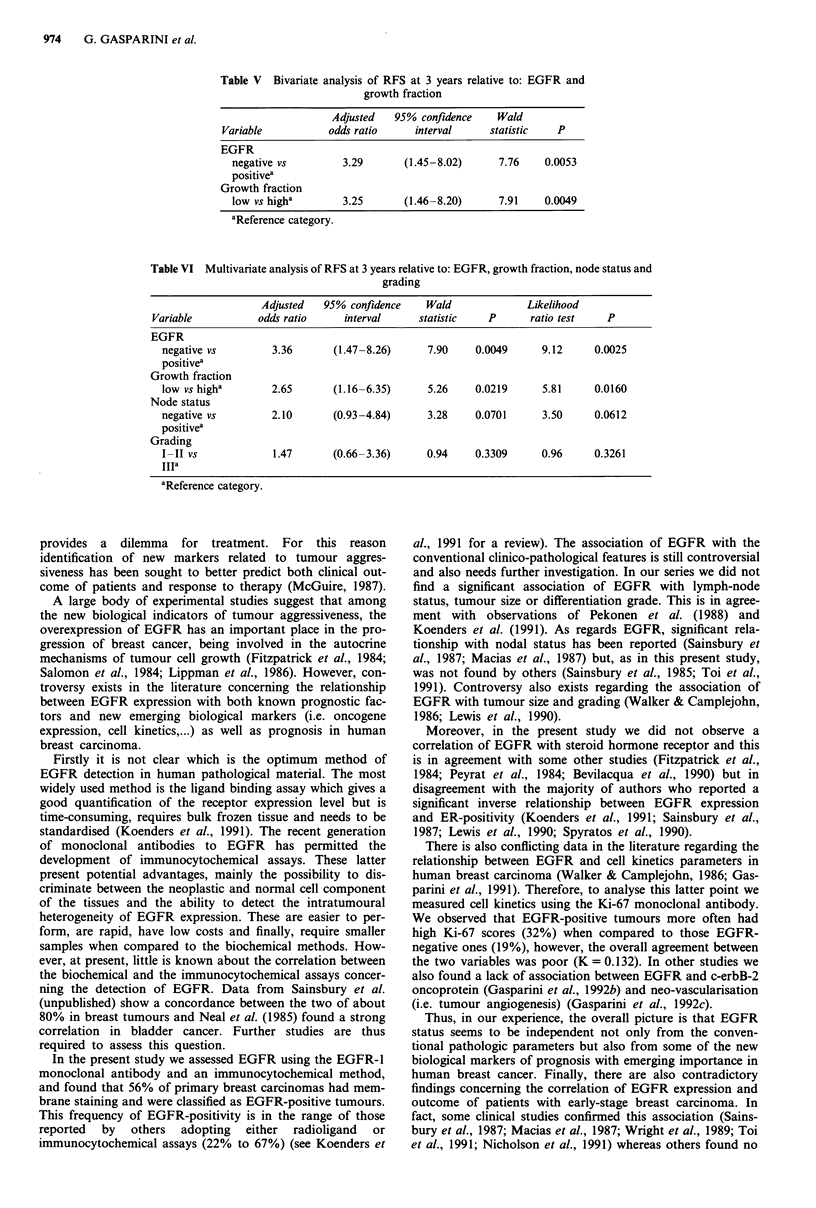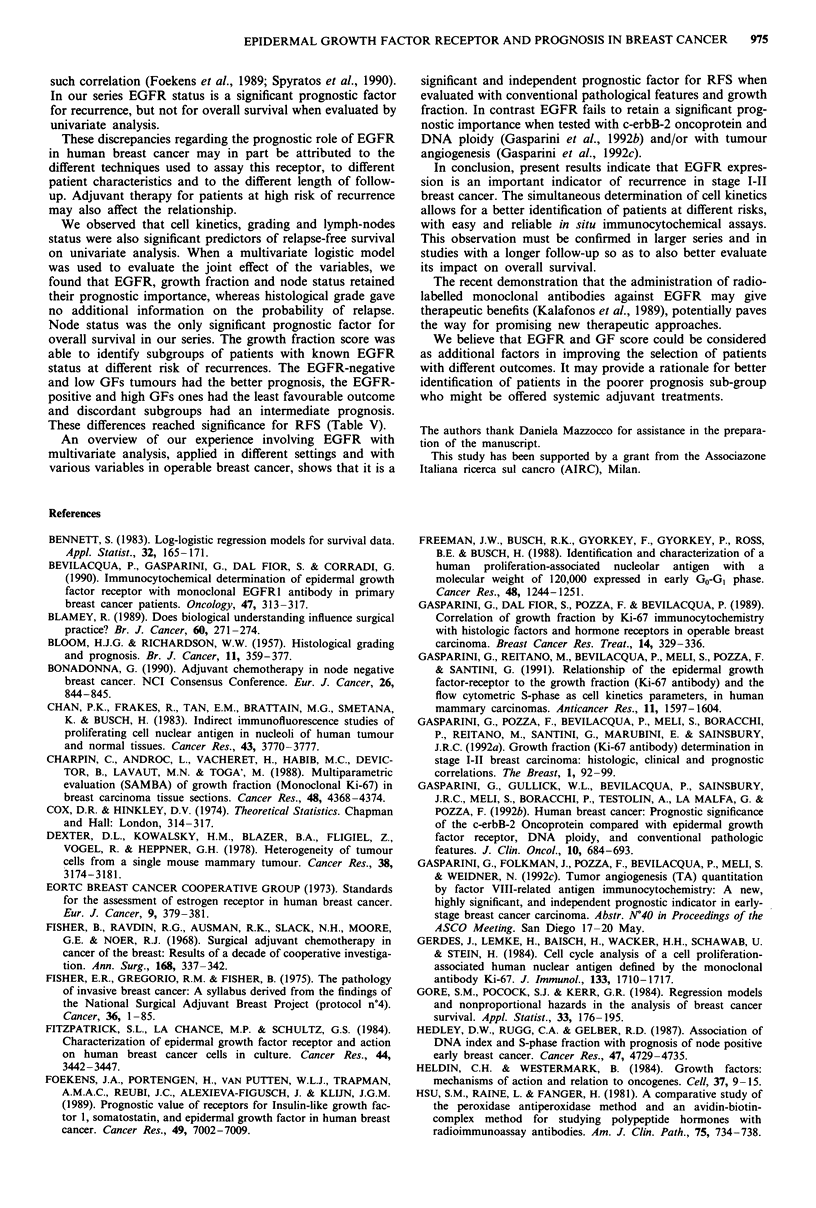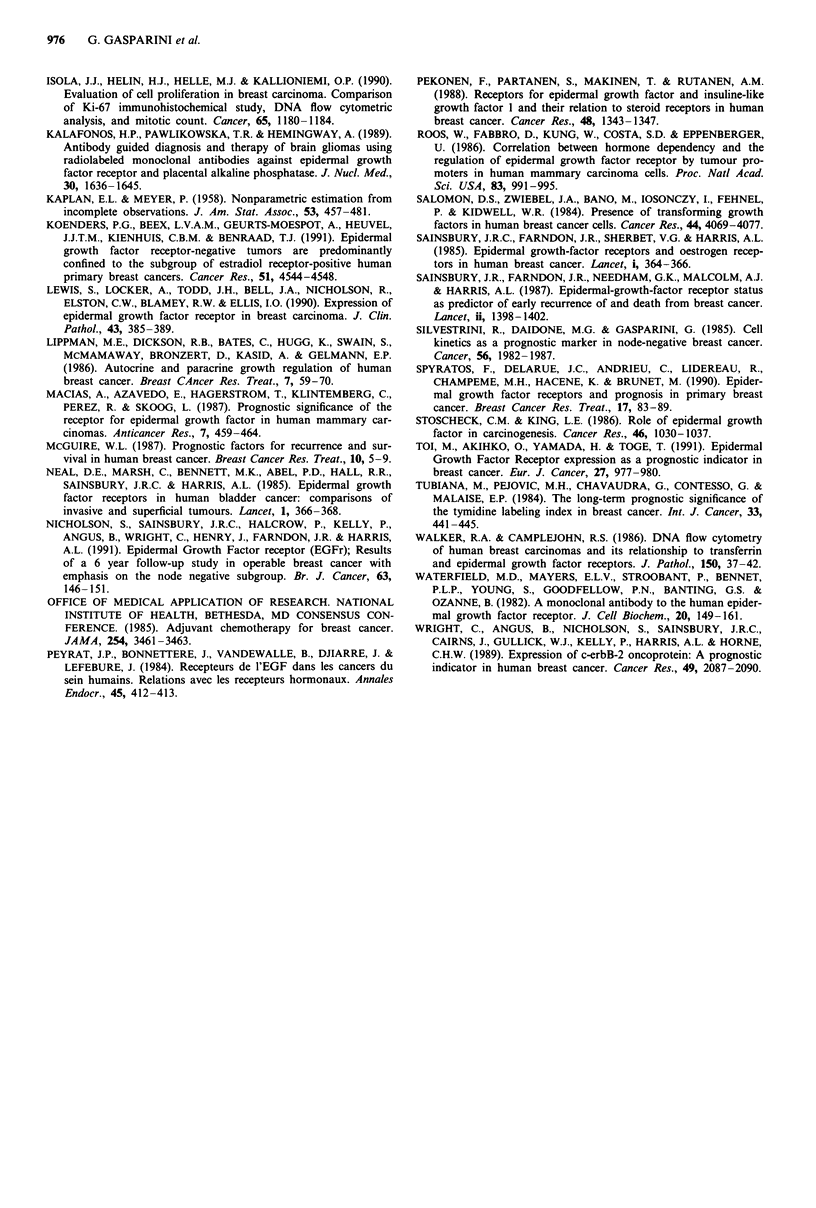# Value of epidermal growth factor receptor status compared with growth fraction and other factors for prognosis in early breast cancer.

**DOI:** 10.1038/bjc.1992.394

**Published:** 1992-11

**Authors:** G. Gasparini, P. Bevilacqua, F. Pozza, S. Meli, P. Boracchi, E. Marubini, J. R. Sainsbury

**Affiliations:** St. Bortolo Regional Hospital, USSL 8 Vicenza-Veneto, Italy.

## Abstract

The epidermal growth factor receptor (EGFR) is a transmembrane glycoprotein whose expression is important in the regulation of breast cancer cell growth. The relationship between EGFR status (determined by an immunocytochemical assay) and various prognostic factors was investigated in 164 primary breast cancers. Overall 56% of tumours were EGFR-positive and the expression of EGFR was unrelated to axillary node status, tumour size and histological grade; and it was poorly associated with the tumour proliferative activity measured by Ki-67 immuno-cytochemistry. The relapse-free survival (RFS) probability at 3-years was significantly worse for patients with EGFR positive tumours (P = 0.003) and for those whose Ki-67 score was > 7.5% (P = 0.0027), as well as in patients with axillary node involvement (P = 0.01) and with poorly differentiated tumours (P = 0.04). Immunocytochemical determination of EGFR and cell kinetics gave superimposable prognostic information for predicting RFS with odds ratios of 3.51, when evaluated singly. In our series of patients EGFR, Ki-67 and node status retain their prognostic value concerning RFS in multivariate analysis. The 3-year probability of overall survival (OS) was significantly better in node-negative patients (P = 0.04) and was similar in EGFR-positive and negative patients. In conclusion, EGFR status appears to be a significant and independent indicator of recurrence in human breast cancer and the concomitant measurement of the tumour proliferative activity seems to improve the selection of patients with different risks of recurrence.


Br. J. Cancer (1992), 66, 970-976 © Macmillan Press Ltd., 1992

### Value of epidermal growth factor receptor status compared with growth fraction and other factors for prognosis in early breast cancer

G. Gasparini[1], P. Bevilacqua[1], F. Pozza[1], S. Meli[1], P. Boracchi[2], E. Marubini[2] & J.R.C. Sainsbury[3]

[1]*St. Bortolo Regional Hospital, USSL 8 Vicenza-Veneto, Italy;* [2]*Biostatistics Institute University of Milan, National Cancer Institute of Milan, Italy;* [3]*Department of Surgery, Huddersfield Royal Infirmary, Huddersfield HD3 3EA, UK.*

**Summary** The epidermal growth factor receptor (EGFR) is a transmembrane glycoprotein whose expression is important in the regulation of breast cancer cell growth. The relationship between EGFR status (determined by an immunocytochemical assay) and various prognostic factors was investigated in 164 primary breast cancers. Overall 56% of tumours were EGFR-positive and the expression of EGFR was unrelated to axillary node status, tumour size and histological grade; and it was poorly associated with the tumour proliferative activity measured by Ki-67 immuno-cytochemistry. The relapse-free survival (RFS) probability at 3-years was significantly worse for patients with EGFR positive tumours ($P = 0.003$) and for those whose Ki-67 score was >7.5% ($P = 0.0027$), as well as in patients with axillary node involvement ($P = 0.01$) and with poorly differentiated tumours ($P = 0.04$). Immunocytochemical determination of EGFR and cell kinetics gave superimposable prognostic information for predicting RFS with odds ratios of 3.51, when evaluated singly. In our series of patients EGFR, Ki-67 and node status retain their prognostic value concerning RFS in multivariate analysis. The 3-year probability of overall survival (OS) was significantly better in node-negative patients ($P = 0.04$) and was similar in EGFR-positive and negative patients.

In conclusion, EGFR status appears to be a significant and independent indicator of recurrence in human breast cancer and the concomitant measurement of the tumour proliferative activity seems to improve the selection of patients with different risks of recurrence.

At present axillary node involvement is the single best prognostic factor in early breast cancer and is used frequently to identify patients eligible for adjuvant treatment (McGuire, 1987; Fisher *et al.*, 1968). Node status *alone*, however, does not fully account for the varied outcome of the patients and we are still unable to accurately predict the clinical course of all patients. Furthermore, node status is not useful in selecting the best treatment for each case (McGuire, 1987; Blamey, 1989). There is thus an increasing need to define new biological markers, associated with tumour aggressiveness, to allow separation of groups of patients with slowly growing tumours who might need no adjuvant therapy from those at high risk.

Recent studies have reported that overexpression of the epidermal growth factor receptor (EGFR) was associated both with enhanced metastatic potential of some breast cancer cell lines (Fitzpatrick *et al.*, 1984; Roos *et al.*, 1986) and with high risk of early recurrence and death in some clinical studies (Sainsbury *et al.*, 1987; Macias *et al.*, 1987; Toi *et al.*, 1991). Although there is no unequivocal evidence that amplification of EGFR is the initial transforming event in human breast carcinoma (Stoscheck & King, 1986; Heldin & Westermark, 1984), an autocrine mechanism is considered as an important step for the independent growth of tumour cells (Lippman *et al.*, 1986; Salomon *et al.*, 1984). Consequently the prognostic significance of EGFR status needs further investigation (Bonadonna, 1990).

There is also increasing evidence that the measurement of cell kinetics is also of considerable prognostic value (Tubiana *et al.*, 1984; Hedley *et al.*, 1987; Silvestrini *et al.*, 1985). The development of monoclonal antibodies which recognises antigens associated with tumour proliferative activity has allowed its determination by *in situ* immunocytochemical assays (Chan *et al.*, 1983; Freeman *et al.*, 1988). These are both easy and rapid to perform and have some technical advantages over other methods (Gasparini *et al.*, 1989). The Ki-67 monoclonal antibody, which is expressed by proliferating cells only (Gerdes *et al.*, 1984), was reported by our group (Gasparini *et al.*, 1989) and others (Charpin *et al.*, 1988; Isola *et al.*, 1990), to be associated with known features of tumour aggressiveness (i.e.: node involvement, high histologic grade, aneuploidy and absence of steroid hormone receptors).

In the present report we have analysed the relationship between EGFR and the proportion of proliferative cells detected by the monoclonal antibody Ki-67 and conventional features of tumour aggressiveness in order to define their prognostic significance in a series of patients with stage I-II breast cancer.

### Patients and methods

One hundred and seventy patients with operable breast carcinoma who underwent surgery at the St. Bortolo Regional Medical Center of Vicenza from 1987 to 1989 were included in the present prospective study.

The eligibility criteria for the inclusion of women with primary breast cancer in this study were: (1) primary $T_1$-$T_3$; $N_{0-2}$; $M_o$, unilateral breast cancer; (2) a tumour specimen obtained between 1987 and 1989 at the time of mastectomy with analysis of EGFR, Growth Fraction (GR) by Ki-67 and the main clinico-pathologic characteristics (menopausal status, pathologic tumour size, node status, and histologic grade); (3) no other primary cancer. All patients were staged according to the UICC-TNM classification.

One hundred and sixty-four of the 170 eligible patients were evaluable, six being lost to follow-up. Of the 164 evaluable patients, 90 underwent radical mastectomy and 74 had quadrantectomy plus radiotherapy. Adjuvant treatments were administered to all node-positive patients according to the recommendations of the 2nd NIH Consensus Development Conference on Adjuvant Chemotherapy and Endocrine Therapy for Breast Cancer (1985). Cyclophosphamide, methotrexate and 5-fluorouracil (CMF) chemotherapy was administered for eight cycles every 21 days in pre-perimenopausal and in ER-negative postmenopausal patients (51 cases); tamoxifen 10 mg t.i.d was given orally daily for 3 years in ER-positive postmenopausal patients (30 cases). Patients who underwent radical mastectomy who were found

Correspondence: G. Gasparini, St Bortolo Regional Hospital, 36100 Vicenza, Italy.
Received 2 October 1991; and in revised form 15 May 1992.

to have more than seven axillary nodes involved, received subsequent adjuvant radiotherapy (32 cases).

Patient characteristics are shown in Table I. At the time of this report, the median follow-up was 36 months (range 12 to 42 months).

Tumour size was recorded as the largest diameter of the tumour at the time of trimming the fresh specimens.

For histologic determination the surgical specimens were first evaluated by frozen section and then fixed in buffered formalin for 24 h at 20°C, dehydrated through graded ethanol and paraffin embedded at 56°C for 30 min. Tumours were classified by histologic type according to the criteria of the National Surgical Adjuvant Breast Project (Fisher *et al.*, 1975).

Histologic grading was according to the criteria of Bloom and Richardson (1957). All identifiable lymph-nodes in the axillary specimens were examined by light microscopy after haematoxylin and eosin staining (median cleared = 16). Biochemical hormonal receptor assay was performed in snap-frozen tumour samples according to the E.O.R.T.C. method (1973). Samples with apparent affinity constants $>0.56 \times 10^9$ mol $l^{-1}$ and $>10$ f.mol $mg^{-1}$ of cytosol protein were considered oestrogen receptor (OER) or progesterone receptor (PgR)-positive. EGFR was analysed with the monoclonal antibody EGFR1, isolated by Waterfield *et al.* (1982) (clone EGFR1 Amersham International Lab, UK). Immunocytochemical staining was carried out by an avidin-biotin complex immunoperoxidase method (Hsu *et al.*, 1981). Serial sections used for cryostatic intraoperative diagnosis were used for immunocytochemistry and the analysis was performed as previously described (Bevilacqua *et al.*, 1990). Tumours were classified as EGFR-positive (at least 5% of cells with membrane staining) or negative, adopting the criteria already published (Bevilacqua *et al.*, 1990). Epidermal growth factor receptor was used as a dichotonomous variable and scored as either positive or negative. Intensity of staining was not considered. Adopting this classification 92 tumours (56%) were EGFR positive while 72 were EGFR negative. The commercially available Dakopatts Kit was used for the GF analysis (Dakopatts Ltd, UK). This employs the IgG1 mouse Moab Ki-67, produced by Gerdes *et al.* (Gerdes *et al.*, 1984).

**Table I** Clinico-pathologic characterstics of the patients

| | *N° of cases* | *%* |
|---|---|---|
| Total evaluable | 164/170 | 95 |
| Median age yrs (range) | 56 (31–71) | – |
| Menopausal status | | |
| premenopausal | 47 | 28.5 |
| perimenopausal | 14 | 8.5 |
| postmenopausal | 103 | 63.0 |
| Tumour size | | |
| pT1 | 93 | 56.5 |
| pT2 | 67 | 41.0 |
| pT3 | 4 | 2.5 |
| Node status | | |
| negative | 83 | 50.5 |
| positive | 81 | 49.5 |
| 1–3 | 46 | |
| 4–7 | 17 | |
| 8–10 | 9 | |
| >10 | 9 | |
| Grading | | |
| I | 10 | 6.0 |
| II | 76 | 46.5 |
| III | 78 | 47.5 |
| Growth fraction score | | |
| High (>7.5%) | 83 | 51 |
| Low (<7.5%) | 81 | 49 |
| [a]Oestrogen receptor status | | |
| negative | 67 | 44 |
| positive | 84 | 56 |
| [a]Progesterone receptor status | | |
| negative | 86 | 57 |
| positive | 65 | 43 |

[a]Available in 150 patients.

The determination of Ki-67 staining was performed as previously reported (Gasparini *et al.*, 1989). Briefly: the immunocytochemical assay was performed on frozen sections (5 um) fixed in cold acetone (– 10°C), washed in 0.01 M phosphate-buffered saline (PBS). Primary antibody was diluted 1:50 for 60 min and tested in biotinylated horse anti-mouse Ig for 30 min (Vector Lab, Burlingame, CA, USA) and avidin-biotinylated horseradish peroxidase complex for 30 min (Vector Lab, Burlingame, CA, USA). A diamino-benzidine hydrogen peroxide substrate was employed as chromogen. The number of cells with nuclear staining was determined in each slide by two observers independently, counting the number of positive nuclei and the total number of nuclei in 12 fields ( × 20 objective, Zeiss photomicroscope). We considered positive those nuclei with diffuse brown nuclear staining.

Since tumours appeared to be heterogeneous for Ki-67 labelling, the determination of GF count was done in the areas of the section with the most intense labelling rate (i.e. at the 'hot spot'). Intensity of staining was not considered. An average of 1,000 nuclei per section were counted. Growth fraction was used as a dichotomous variable, and the median value of 7.5% was used as cut-off point to discriminate slowly versus rapidly proliferating tumours. Adopting this criterion, 81 tumours (49%) were classified as slowly proliferating (Ki-67 scores <7.5%) while 83 (51%) as rapidly proliferating tumours (Ki-67 scores >7.5%).

All patients were followed-up postoperatively and physical examination was performed monthly during the treatment with adjuvant CMF or the first 6 months of adjuvant tamoxifen in node-positive cases. In these women, after completion of the adjuvant programme, as well as in all node-negative women, physical examination was performed every 4 months during the first 3 years following surgery. Six patients were lost to follow-up and were not included in the analysis of prognostic factors. Primary treatment failure was defined as the first documented evidence of new disease manifestation(s) in loco-regional area(s), distant site(s), contralateral breast, or a combination of the above. Any new disease involvement was assessed by clinical, radiological and, wherever feasible, histologic examination of the site(s) of first relapse.

### *Statistics*

The association between EGFR and the clinico-pathologic variables was evaluated by Chi-square ($\chi^2$) test. The agreement between EGFR and GF was analysed by the K coefficient (Table II). The patterns of overall survival (OS) and relapse-free survival (RFS) were estimated by means of the product-limit method (Kaplan & Meyer, 1958). Preliminary graphical analyses suggested that the proportional hazard assumption was not tenable; on the other hand, the plots of log (odds) (probability of surviving/probability of dying) against log (time) for all the categories of prognostic variables resulted in parallel straight lines. Therefore the role of each of the prognostic variables (univariate analysis) and their joint effect (multivariate analysis) were evaluated using a log-logistic regression model (Bennett, 1983). This was shown to be suitable for fitting breast cancer data accumulated from previous reports (Gore *et al.*, 1984). In the log-logistic regression model each of the regression coefficients ($\beta$) is recognisable as the log (odds ratio = OR) and is constant in time (Bennett, 1983). For patients classified in two prognostic categories and having the same survival experience, the statis-

**Table II** Association between EGFR and cell kinetics by Ki-67 immunostaining

| *EGFR* | *Ki-67 score* | | |
|---|---|---|---|
| | low (<7.5%) | high (>7.5%) | Total |
| Negative | 41 (25%) | 31 (19%) | 72 |
| Positive | 40 (24%) | 52 (32%) | 92 |
| Total | 81 | 83 | 164 |

K coefficient = 0.132.

**Table III** Distribution of the clinico-pathological characteristics according to the EGFR expression

| Feature | EGFR Positive n (%) | EGFR Negative n (%) | $\chi^2$ | DF | P |
|---|---|---|---|---|---|
| Overall | 92 (56) | 72 (44) | – | – | – |
| Menopausal status | | | | | |
| Premenopausal | 25 (27) | 22 (31) | | | |
| Perimenopausal | 10 (11) | 4 (5) | 1.52 | 2 | 0.467 |
| Postmenopausal | 57 (62) | 46 (64) | | | |
| Tumour size | | | | | |
| pT1 | 49 (53) | 44 (61) | | | |
| pT2-3 | 43 (47) | 28 (39) | 1.01 | 1 | 0.314 |
| Node status | | | | | |
| negative | 46 (50) | 37 (51) | | | |
| positive | 46 (50) | 35 (49) | 0.03 | 1 | 0.860 |
| Grading | | | | | |
| I-II | 46 (50) | 40 (56) | | | |
| III | 46 (50) | 32 (44) | 0.50 | 1 | 0.480 |
| Oestrogen receptor status[a] | | | | | |
| negative | 38 (46) | 29 (42) | | | |
| positive | 44 (54) | 40 (58) | 0.28 | 1 | 0.595 |
| Progesterone receptor status[a] | | | | | |
| negative | 44 (54) | 42 (61) | | | |
| positive | 38 (46) | 27 (39) | 0.79 | 1 | 0.373 |

[a]14 pts had missing information about hormonal receptors DF = degrees of freedom.

tic : exp($\beta$) = 0 is expected to be 1.0. For OR < 1 (OR > 1) patients classified in a given category have an odds of surviving lower (greater) than that of patients in the reference category. The hypothesis B = 0 was teted by Wald statistic (Cox & Hinkley, 1974). For each variable, 'unadjusted' odds ratios and their 95% confidence intervals were estimated using the putative 'poorest prognosis' class as reference category. To investigate the prognostic relevance of EGFR allowing for the other prognostic variables the 'adjusted' odds ratios were estimated using a multiple regression model containing, besides EGFR, those variables which had unadjusted odds ratios significantly different from 1.0.

The impact of each prognostic factor on clinical outcome, in addition to that of the remaining variables, was evaluated by means of the likelihood ratio statistic.

### Results

### *Immunocytochemical staining with the EGFR1 monoclonal antibody*

EGFR1 antibody produced immunostaining labelling of variable intensity and extent in the *membrane* of tumour cells in 92 carcinomas (56%). Seventy-two tumours had only a weak focal or failed to retain any convincing membrane staining. This frequency of EGFR positivity is in accordance with that previously found by our group (Bevilacqua *et al.*, 1990) and by others (Sainsbury *et al.*, 1985). All slides were independently evaluated by two investigators (P.B.; S.M.).

### *Correlation of EGFR to other prognostic indicators*

One hundred and forty-four of 164 tumours (88%) had stained *nuclei* with GF scores varying from 1% to 80% (median value = 7.5%) using the Ki-67 antibody. We observed that EGFR-positive tumours had a slightly higher GF score when compared to those EGFR-negative (K coefficient = 0.132). The median average proportion of Ki-67 stained cells in EGFR-positive tumours was 10% compared to 5% in the EGFR-negative ones. As shown in Table II, GF was able to identify high proliferative activity within both EGFR-positive and EGFR-negative tumours. As reported in Table III the expression of EGFR was not significantly associated with the other clinico-pathologic features analysed.

### *Clinical results*

After a median follow-up of the patients of 36 months, the overall 3-year probability of OS and RFS was 83% and 73%, respectively.

During the follow-up period 40 patients had recurrence of disease. Thirty-three of them presented with distant metastases, six developed metachronous breast carcinoma and one had an isolated local relapse.

Twenty-four patients died, eight from causes unrelated to breast carcinoma (included in the analysis).

### *Univariate analysis*

*Prognostic significance of the epidermal growth factor receptor* The survival analysis suggested that the EGFR positivity was significantly associated with recurrence. The 3-year probability of RFS was 83% for patients with EGFR-negative tumours (10/72 patients relapsed) compared to 65% for patients with EGFR-positive tumours (30/92 patients relapsed). EGFR-negative conferred a significantly lower probability of relapse compared to the EGFR-positive ones with an odds of not relapsing of 3.51 ($\chi^2 = 8.72$, $P = 0.003$) (Table IV) (Figure 1). The 3-year probability of OS was similar in patients with EGFR-negative tumours compared to the EGFR-positive ones (85% *vs* 81%) with an odds ratio of 1.09 (Table IV).

*Prognostic Significance of the other factors* In addition to EGFR expression we investigated the prognostic importance of GF using Ki-67 antibody, lymph node status, histologic grade, pathologic tumour size and menopausal status.

We found that patients with low GF scores (< 7.5%) had a significantly lower probability of recurrence with an odds of not relapsing of 3.51 ($\chi^2 = 8.98$, $P = 0.0027$) compared to those with high GFs (> 7.5%) whereas patients with low GF scores had only a slightly higher survival when compared to those with high proliferating tumours (odds ratio of 1.79). More details concerning the clinical results on GF have been reported elsewhere (Gasparini *et al.*, 1992*a*). When patients were divided into four subsets on the basis of EGFR status and GF score, the subset of patients with EGFR-positive tumours and with high GF scores had the poorest 3-year RFS rate whereas the subgroup of those with EGFR-negative tumours and low GF scores had the best RFS (Figure 2). Bivariate analysis showed that the difference

**Table IV** Univariate analysis of RFS and OS at 3 years

| | RFS | | | | OS | | | |
|---|---|---|---|---|---|---|---|---|
| *Variable* | *Unadjusted odds ratio* | *95% confidence interval* | *Wald statistic* | P | *Unadjusted odds ratio* | *95% confidence interval* | *Wald statistic* | P |
| EGFR | | | | | | | | |
| negative *vs* positive[a] | 3.51 | (1.57–8.41) | 8.72 | 0.0031 | 1.09 | (0.42–2.92) | 0.04 | 0.8430 |
| Growth fraction | | | | | | | | |
| low *vs* high[a] | 3.51 | (1.59–8.20) | 8.99 | 0.0027 | 1.79 | (0.69–4.77) | 1.57 | 0.2091 |
| Node status | | | | | | | | |
| negative *vs* positive[a] | 2.59 | (1.21–5.76) | 5.81 | 0.0159 | 2.79 | (1.03–7.93) | 4.06 | 0.0439 |
| Grading | | | | | | | | |
| I–II *vs* III[a] | 2.19 | 1.04–4.74) | 4.21 | 0.0401 | 1.34 | (0.53–3.46) | 0.43 | 0.5135 |
| Tumour size | | | | | | | | |
| $pT_1$ *vs* $pT_{2/3}$[a] | 1.53 | (0.74–3.27) | 1.38 | 0.2407 | 1.76 | (0.71–4.67) | 1.59 | 0.2078 |
| Menopausal status | | | | | | | | |
| postmenopausal *vs* premenopausal[a] | 0.91 | (0.39–2.11) | 0.05 | 0.8237 | 0.63 | (0.19–1.96) | 0.72 | 0.3958 |
| perimenopausal *vs* premenopausal[a] | 0.44 | (0.12–1.48) | 1.81 | 0.1784 | 0.40 | (0.07–2.11) | 1.25 | 0.2625 |
| OER | | | | | | | | |
| negative[a] *vs* positive | 1.79 | (0.81–3.99) | 2.16 | 0.1418 | 0.68 | (0.22–1.85) | 0.63 | 0.4265 |
| PGR | | | | | | | | |
| negative[a] *vs* positive | 0.90 | (0.40–1.99) | 0.07 | 0.7940 | 1.56 | (0.51–4.78) | 0.70 | 0.4000 |

[a]Reference category.

between subgroups reached significance (Table V). Patients with poorly differentiated tumours had a significantly higher frequency of recurrence ($\chi^2 = 4.21$; $P = 0.040$), but the difference did not reach significance concerning OS. Pathologic tumour size, menopausal status and steroid hormone receptors did not significantly influence the outcome of patients concerning both RFS and OS.

Finally, node status was the only factor that in the present series significantly influenced both RFS and OS within 3-years of surgery (Table IV).

*Multivariate prognostic analysis*

To evaluate the joint effect of the variables analysed, only the terms relative to the main effects were inserted into the final model as far as RFS was concerned. Since only node status was significantly related with OS we did not perform a multivariate analysis at this level.

GF, grading and node status were introduced first into the model and EGFR was then added. A large increase (likelihood ratio test, $\chi^2 = 9.12$) was observed on introduction of EGFR. When GF was added to the model containing node status and EGFR a significant contribution of GF in identifying patients with different risk was seen (likelihood ratio test, $\chi^2 = 5.81$). Finally when grading was added to the above variables, a non significant likelihood ratio test was obtained ($\chi^2 = 0.96$; $P > 0.05$). This suggests that grading gives no additional information on prognosis when the patients are already classified by the other three factors (Table VI). The contribution of node status was nearly significant, when all the four variables were considered and reached significance in a model that does not include grading (likelihood ratio test $\chi^2 = 4.42$; $P = 0.035$).

**Figure 1** 3-year probability of relapse-free survival (RFS) in EGFR-positive (– – – –) and EGFR-negative (———) patients. Patients at risk at 3 years: EGFR-positive = 15; EGFR-negative = 25.

### Discussion

Human breast cancer is a neoplasia characterised by a substantial heterogeneity, with various clones of cells of differing growth and metastatic potential (Dexter *et al.*, 1978) which

**Figure 2** 3-year probability of relapse-free survival (RFS) in the four subsets of patients on the basis of the EGFR status and GF score: EGFR-positive and high GF score (52 patients) (– – – –); EGFR-negative and high GF score (31 patients (....); EGFR-positive and low GF score (40 patients) (——); and EGFR-negative and low GF score (41 patients) (——). Patients at risk at 3-years: EGFR-positive and high GF = 4; EGFR-negative and high GF = 11; EGFR-positive and low GF = 8 and EGFR-negative and low GF = 41.

**Table V** Bivariate analysis of RFS at 3 years relative to: EGFR and growth fraction

| *Variable* | *Adjusted odds ratio* | *95% confidence interval* | *Wald statistic* | *P* |
|---|---|---|---|---|
| EGFR | | | | |
| negative *vs* positive[a] | 3.29 | (1.45–8.02) | 7.76 | 0.0053 |
| Growth fraction | | | | |
| low *vs* high[a] | 3.25 | (1.46–8.20) | 7.91 | 0.0049 |

[a]Reference category.

**Table VI** Multivariate analysis of RFS at 3 years relative to: EGFR, growth fraction, node status and grading

| *Variable* | *Adjusted odds ratio* | *95% confidence interval* | *Wald statistic* | *P* | *Likelihood ratio test* | *P* |
|---|---|---|---|---|---|---|
| EGFR | | | | | | |
| negative *vs* positive[a] | 3.36 | (1.47–8.26) | 7.90 | 0.0049 | 9.12 | 0.0025 |
| Growth fraction | | | | | | |
| low *vs* high[a] | 2.65 | (1.16–6.35) | 5.26 | 0.0219 | 5.81 | 0.0160 |
| Node status | | | | | | |
| negative *vs* positive[a] | 2.10 | (0.93–4.84) | 3.28 | 0.0701 | 3.50 | 0.0612 |
| Grading | | | | | | |
| I–II *vs* III[a] | 1.47 | (0.66–3.36) | 0.94 | 0.3309 | 0.96 | 0.3261 |

[a]Reference category.

provides a dilemma for treatment. For this reason identification of new markers related to tumour aggressiveness has been sought to better predict both clinical outcome of patients and response to therapy (McGuire, 1987).

A large body of experimental studies suggest that among the new biological indicators of tumour aggressiveness, the overexpression of EGFR has an important place in the progression of breast cancer, being involved in the autocrine mechanisms of tumour cell growth (Fitzpatrick *et al.*, 1984; Salomon *et al.*, 1984; Lippman *et al.*, 1986). However, controversy exists in the literature concerning the relationship between EGFR expression with both known prognostic factors and new emerging biological markers (i.e. oncogene expression, cell kinetics,...) as well as prognosis in human breast carcinoma.

Firstly it is not clear which is the optimum method of EGFR detection in human pathological material. The most widely used method is the ligand binding assay which gives a good quantification of the receptor expression level but is time-consuming, requires bulk frozen tissue and needs to be standardised (Koenders *et al.*, 1991). The recent generation of monoclonal antibodies to EGFR has permitted the development of immunocytochemical assays. These latter present potential advantages, mainly the possibility to discriminate between the neoplastic and normal cell component of the tissues and the ability to detect the intratumoural heterogeneity of EGFR expression. These are easier to perform, are rapid, have low costs and finally, require smaller samples when compared to the biochemical methods. However, at present, little is known about the correlation between the biochemical and the immunocytochemical assays concerning the detection of EGFR. Data from Sainsbury *et al.* (unpublished) show a concordance between the two of about 80% in breast tumours and Neal *et al.* (1985) found a strong correlation in bladder cancer. Further studies are thus required to assess this question.

In the present study we assessed EGFR using the EGFR-1 monoclonal antibody and an immunocytochemical method, and found that 56% of primary breast carcinomas had membrane staining and were classified as EGFR-positive tumours. This frequency of EGFR-positivity is in the range of those reported by others adopting either radioligand or immunocytochemical assays (22% to 67%) (see Koenders *et al.*, 1991 for a review). The association of EGFR with the conventional clinico-pathological features is still controversial and also needs further investigation. In our series we did not find a significant association of EGFR with lymph-node status, tumour size or differentiation grade. This is in agreement with observations of Pekonen *et al.* (1988) and Koenders *et al.* (1991). As regards EGFR, significant relationship with nodal status has been reported (Sainsbury *et al.*, 1987; Macias *et al.*, 1987) but, as in this present study, was not found by others (Sainsbury *et al.*, 1985; Toi *et al.*, 1991). Controversy also exists regarding the association of EGFR with tumour size and grading (Walker & Camplejohn, 1986; Lewis *et al.*, 1990).

Moreover, in the present study we did not observe a correlation of EGFR with steroid hormone receptor and this is in agreement with some other studies (Fitzpatrick *et al.*, 1984; Peyrat *et al.*, 1984; Bevilacqua *et al.*, 1990) but in disagreement with the majority of authors who reported a significant inverse relationship between EGFR expression and ER-positivity (Koenders *et al.*, 1991; Sainsbury *et al.*, 1987; Lewis *et al.*, 1990; Spyratos *et al.*, 1990).

There is also conflicting data in the literature regarding the relationship between EGFR and cell kinetics parameters in human breast carcinoma (Walker & Camplejohn, 1986; Gasparini *et al.*, 1991). Therefore, to analyse this latter point we measured cell kinetics using the Ki-67 monoclonal antibody. We observed that EGFR-positive tumours more often had high Ki-67 scores (32%) when compared to those EGFR-negative ones (19%), however, the overall agreement between the two variables was poor (K = 0.132). In other studies we also found a lack of association between EGFR and c-erbB-2 oncoprotein (Gasparini *et al.*, 1992*b*) and neo-vascularisation (i.e. tumour angiogenesis) (Gasparini *et al.*, 1992*c*).

Thus, in our experience, the overall picture is that EGFR status seems to be independent not only from the conventional pathologic parameters but also from some of the new biological markers of prognosis with emerging importance in human breast cancer. Finally, there are also contradictory findings concerning the correlation of EGFR expression and outcome of patients with early-stage breast carcinoma. In fact, some clinical studies confirmed this association (Sainsbury *et al.*, 1987; Macias *et al.*, 1987; Wright *et al.*, 1989; Toi *et al.*, 1991; Nicholson *et al.*, 1991) whereas others found no such correlation (Foekens *et al.*, 1989; Spyratos *et al.*, 1990). In our series EGFR status is a significant prognostic factor for recurrence, but not for overall survival when evaluated by univariate analysis.

These discrepancies regarding the prognostic role of EGFR in human breast cancer may in part be attributed to the different techniques used to assay this receptor, to different patient characteristics and to the different length of follow-up. Adjuvant therapy for patients at high risk of recurrence may also affect the relationship.

We observed that cell kinetics, grading and lymph-nodes status were also significant predictors of relapse-free survival on univariate analysis. When a multivariate logistic model was used to evaluate the joint effect of the variables, we found that EGFR, growth fraction and node status retained their prognostic importance, whereas histological grade gave no additional information on the probability of relapse. Node status was the only significant prognostic factor for overall survival in our series. The growth fraction score was able to identify subgroups of patients with known EGFR status at different risk of recurrences. The EGFR-negative and low GFs tumours had the better prognosis, the EGFR-positive and high GFs ones had the least favourable outcome and discordant subgroups had an intermediate prognosis. These differences reached significance for RFS (Table V).

An overview of our experience involving EGFR with multivariate analysis, applied in different settings and with various variables in operable breast cancer, shows that it is a significant and independent prognostic factor for RFS when evaluated with conventional pathological features and growth fraction. In contrast EGFR fails to retain a significant prognostic importance when tested with c-erbB-2 oncoprotein and DNA ploidy (Gasparini *et al.*, 1992*b*) and/or with tumour angiogenesis (Gasparini *et al.*, 1992*c*).

In conclusion, present results indicate that EGFR expression is an important indicator of recurrence in stage I-II breast cancer. The simultaneous determination of cell kinetics allows for a better identification of patients at different risks, with easy and reliable *in situ* immunocytochemical assays. This observation must be confirmed in larger series and in studies with a longer follow-up so as to also better evaluate its impact on overall survival.

The recent demonstration that the administration of radio-labelled monoclonal antibodies against EGFR may give therapeutic benefits (Kalafonos *et al.*, 1989), potentially paves the way for promising new therapeutic approaches.

We believe that EGFR and GF score could be considered as additional factors in improving the selection of patients with different outcomes. It may provide a rationale for better identification of patients in the poorer prognosis sub-group who might be offered systemic adjuvant treatments.

The authors thank Daniela Mazzocco for assistance in the preparation of the manuscript.

This study has been supported by a grant from the Associazone Italiana ricerca sul cancro (AIRC), Milan.

### References

BENNETT, S. (1983). Log-logistic regression models for survival data. *Appl. Statist.*, **32**, 165-171.

BEVILACQUA, P., GASPARINI, G., DAL FIOR, S. & CORRADI, G. (1990). Immunocytochemical determination of epidermal growth factor receptor with monoclonal EGFR1 antibody in primary breast cancer patients. *Oncology*, **47**, 313-317.

BLAMEY, R. (1989). Does biological understanding influence surgical practice? *Br. J. Cancer*, **60**, 271-274.

BLOOM, H.J.G. & RICHARDSON, W.W. (1957). Histological grading and prognosis. *Br. J. Cancer*, **11**, 359-377.

BONADONNA, G. (1990). Adjuvant chemotherapy in node negative breast cancer. NCI Consensus Conference. *Eur. J. Cancer*, **26**, 844-845.

CHAN, P.K., FRAKES, R., TAN, E.M., BRATTAIN, M.G., SMETANA, K. & BUSCH, H. (1983). Indirect immunofluorescence studies of proliferating cell nuclear antigen in nucleoli of human tumour and normal tissues. *Cancer Res.*, **43**, 3770-3777.

CHARPIN, C., ANDROC, L., VACHERET, H., HABIB, M.C., DEVICTOR, B., LAVAUT, M.N. & TOGA', M. (1988). Multiparametric evaluation (SAMBA) of growth fraction (Monoclonal Ki-67) in breast carcinoma tissue sections. *Cancer Res.*, **48**, 4368-4374.

COX, D.R. & HINKLEY, D.V. (1974). *Theoretical Statistics*. Chapman and Hall: London, 314-317.

DEXTER, D.L., KOWALSKY, H.M., BLAZER, B.A., FLIGIEL, Z., VOGEL, R. & HEPPNER, G.H. (1978). Heterogeneity of tumour cells from a single mouse mammary tumour. *Cancer Res.*, **38**, 3174-3181.

EORTC BREAST CANCER COOPERATIVE GROUP (1973). Standards for the assessment of estrogen receptor in human breast cancer. *Eur. J. Cancer*, **9**, 379-381.

FISHER, B., RAVDIN, R.G., AUSMAN, R.K., SLACK, N.H., MOORE, G.E. & NOER, R.J. (1968). Surgical adjuvant chemotherapy in cancer of the breast: Results of a decade of cooperative investigation. *Ann. Surg.*, **168**, 337-342.

FISHER, E.R., GREGORIO, R.M. & FISHER, B. (1975). The pathology of invasive breast cancer: A syllabus derived from the findings of the National Surgical Adjuvant Breast Project (protocol n°4). *Cancer*, **36**, 1-85.

FITZPATRICK, S.L., LA CHANCE, M.P. & SCHULTZ, G.S. (1984). Characterization of epidermal growth factor receptor and action on human breast cancer cells in culture. *Cancer Res.*, **44**, 3442-3447.

FOEKENS, J.A., PORTENGEN, H., VAN PUTTEN, W.L.J., TRAPMAN, A.M.A.C., REUBI, J.C., ALEXIEVA-FIGUSCH, J. & KLIJN, J.G.M. (1989). Prognostic value of receptors for Insulin-like growth factor 1, somatostatin, and epidermal growth factor in human breast cancer. *Cancer Res.*, **49**, 7002-7009.

FREEMAN, J.W., BUSCH, R.K., GYORKEY, F., GYORKEY, P., ROSS, B.E. & BUSCH, H. (1988). Identification and characterization of a human proliferation-associated nucleolar antigen with a molecular weight of 120,000 expressed in early $G_0$-$G_1$ phase. *Cancer Res.*, **48**, 1244-1251.

GASPARINI, G., DAL FIOR, S., POZZA, F. & BEVILACQUA, P. (1989). Correlation of growth fraction by Ki-67 immunocytochemistry with histologic factors and hormone receptors in operable breast carcinoma. *Breast Cancer Res. Treat.*, **14**, 329-336.

GASPARINI, G., REITANO, M., BEVILACQUA, P., MELI, S., POZZA, F. & SANTINI, G. (1991). Relationship of the epidermal growth factor-receptor to the growth fraction (Ki-67 antibody) and the flow cytometric S-phase as cell kinetics parameters, in human mammary carcinomas. *Anticancer Res.*, **11**, 1597-1604.

GASPARINI, G., POZZA, F., BEVILACQUA, P., MELI, S., BORACCHI, P., REITANO, M., SANTINI, G., MARUBINI, E. & SAINSBURY, J.R.C. (1992*a*). Growth fraction (Ki-67 antibody) determination in stage I-II breast carcinoma: histologic, clinical and prognostic correlations. *The Breast*, **1**, 92-99.

GASPARINI, G., GULLICK, W.L., BEVILACQUA, P., SAINSBURY, J.R.C., MELI, S., BORACCHI, P., TESTOLIN, A., LA MALFA, G. & POZZA, F. (1992*b*). Human breast cancer: Prognostic significance of the c-erbB-2 Oncoprotein compared with epidermal growth factor receptor, DNA ploidy, and conventional pathologic features. *J. Clin. Oncol.*, **10**, 684-693.

GASPARINI, G., FOLKMAN, J., POZZA, F., BEVILACQUA, P., MELI, S. & WEIDNER, N. (1992*c*). Tumor angiogenesis (TA) quantitation by factor VIII-related antigen immunocytochemistry: A new, highly significant, and independent prognostic indicator in early-stage breast cancer carcinoma. *Abstr. N°40 in Proceedings of the ASCO Meeting*. San Diego 17-20 May.

GERDES, J., LEMKE, H., BAISCH, H., WACKER, H.H., SCHAWAB, U. & STEIN, H. (1984). Cell cycle analysis of a cell proliferation-associated human nuclear antigen defined by the monoclonal antibody Ki-67. *J. Immunol.*, **133**, 1710-1717.

GORE, S.M., POCOCK, S.J. & KERR, G.R. (1984). Regression models and nonproportional hazards in the analysis of breast cancer survival. *Appl. Statist.*, **33**, 176-195.

HEDLEY, D.W., RUGG, C.A. & GELBER, R.D. (1987). Association of DNA index and S-phase fraction with prognosis of node positive early breast cancer. *Cancer Res.*, **47**, 4729-4735.

HELDIN, C.H. & WESTERMARK, B. (1984). Growth factors: mechanisms of action and relation to oncogenes. *Cell*, **37**, 9-15.

HSU, S.M., RAINE, L. & FANGER, H. (1981). A comparative study of the peroxidase antiperoxidase method and an avidin-biotin-complex method for studying polypeptide hormones with radioimmunoassay antibodies. *Am. J. Clin. Path.*, **75**, 734-738.

ISOLA, J.J., HELIN, H.J., HELLE, M.J. & KALLIONIEMI, O.P. (1990). Evaluation of cell proliferation in breast carcinoma. Comparison of Ki-67 immunohistochemical study, DNA flow cytometric analysis, and mitotic count. *Cancer*, **65**, 1180–1184.

KALAFONOS, H.P., PAWLIKOWSKA, T.R. & HEMINGWAY, A. (1989). Antibody guided diagnosis and therapy of brain gliomas using radiolabeled monoclonal antibodies against epidermal growth factor receptor and placental alkaline phosphatase. *J. Nucl. Med.*, **30**, 1636–1645.

KAPLAN, E.L. & MEYER, P. (1958). Nonparametric estimation from incomplete observations. *J. Am. Stat. Assoc.*, **53**, 457–481.

KOENDERS, P.G., BEEX, L.V.A.M., GEURTS-MOESPOT, A., HEUVEL, J.J.T.M., KIENHUIS, C.B.M. & BENRAAD, T.J. (1991). Epidermal growth factor receptor-negative tumors are predominantly confined to the subgroup of estradiol receptor-positive human primary breast cancers. *Cancer Res.*, **51**, 4544–4548.

LEWIS, S., LOCKER, A., TODD, J.H., BELL, J.A., NICHOLSON, R., ELSTON, C.W., BLAMEY, R.W. & ELLIS, I.O. (1990). Expression of epidermal growth factor receptor in breast carcinoma. *J. Clin. Pathol.*, **43**, 385–389.

LIPPMAN, M.E., DICKSON, R.B., BATES, C., HUGG, K., SWAIN, S., MCMAMAWAY, BRONZERT, D., KASID, A. & GELMANN, E.P. (1986). Autocrine and paracrine growth regulation of human breast cancer. *Breast CAncer Res. Treat.*, **7**, 59–70.

MACIAS, A., AZAVEDO, E., HAGERSTROM, T., KLINTEMBERG, C., PEREZ, R. & SKOOG, L. (1987). Prognostic significance of the receptor for epidermal growth factor in human mammary carcinomas. *Anticancer Res.*, **7**, 459–464.

MCGUIRE, W.L. (1987). Prognostic factors for recurrence and survival in human breast cancer. *Breast Cancer Res. Treat.*, **10**, 5–9.

NEAL, D.E., MARSH, C., BENNETT, M.K., ABEL, P.D., HALL, R.R., SAINSBURY, J.R.C. & HARRIS, A.L. (1985). Epidermal growth factor receptors in human bladder cancer: comparisons of invasive and superficial tumours. *Lancet*, **1**, 366–368.

NICHOLSON, S., SAINSBURY, J.R.C., HALCROW, P., KELLY, P., ANGUS, B., WRIGHT, C., HENRY, J., FARNDON, J.R. & HARRIS, A.L. (1991). Epidermal Growth Factor receptor (EGFr); Results of a 6 year follow-up study in operable breast cancer with emphasis on the node negative subgroup. *Br. J. Cancer*, **63**, 146–151.

OFFICE OF MEDICAL APPLICATION OF RESEARCH. NATIONAL INSTITUTE OF HEALTH, BETHESDA, MD CONSENSUS CONFERENCE. (1985). Adjuvant chemotherapy for breast cancer. *JAMA*, **254**, 3461–3463.

PEYRAT, J.P., BONNETTERE, J., VANDEWALLE, B., DJIARRE, J. & LEFEBURE, J. (1984). Recepteurs de l'EGF dans les cancers du sein humains. Relations avec les recepteurs hormonaux. *Annales Endocr.*, **45**, 412–413.

PEKONEN, F., PARTANEN, S., MAKINEN, T. & RUTANEN, A.M. (1988). Receptors for epidermal growth factor and insuline-like growth factor 1 and their relation to steroid receptors in human breast cancer. *Cancer Res.*, **48**, 1343–1347.

ROOS, W., FABBRO, D., KUNG, W., COSTA, S.D. & EPPENBERGER, U. (1986). Correlation between hormone dependency and the regulation of epidermal growth factor receptor by tumour promoters in human mammary carcinoma cells. *Proc. Natl Acad. Sci. USA*, **83**, 991–995.

SALOMON, D.S., ZWIEBEL, J.A., BANO, M., IOSONCZY, I., FEHNEL, P. & KIDWELL, W.R. (1984). Presence of transforming growth factors in human breast cancer cells. *Cancer Res.*, **44**, 4069–4077.

SAINSBURY, J.R.C., FARNDON, J.R., SHERBET, V.G. & HARRIS, A.L. (1985). Epidermal growth-factor receptors and oestrogen receptors in human breast cancer. *Lancet*, **i**, 364–366.

SAINSBURY, J.R., FARNDON, J.R., NEEDHAM, G.K., MALCOLM, A.J. & HARRIS, A.L. (1987). Epidermal-growth-factor receptor status as predictor of early recurrence of and death from breast cancer. *Lancet*, **ii**, 1398–1402.

SILVESTRINI, R., DAIDONE, M.G. & GASPARINI, G. (1985). Cell kinetics as a prognostic marker in node-negative breast cancer. *Cancer*, **56**, 1982–1987.

SPYRATOS, F., DELARUE, J.C., ANDRIEU, C., LIDEREAU, R., CHAMPEME, M.H., HACENE, K. & BRUNET, M. (1990). Epidermal growth factor receptors and prognosis in primary breast cancer. *Breast Cancer Res. Treat.*, **17**, 83–89.

STOSCHECK, C.M. & KING, L.E. (1986). Role of epidermal growth factor in carcinogenesis. *Cancer Res.*, **46**, 1030–1037.

TOI, M., AKIHKO, O., YAMADA, H. & TOGE, T. (1991). Epidermal Growth Factor Receptor expression as a prognostic indicator in breast cancer. *Eur. J. Cancer*, **27**, 977–980.

TUBIANA, M., PEJOVIC, M.H., CHAVAUDRA, G., CONTESSO, G. & MALAISE, E.P. (1984). The long-term prognostic significance of the tymidine labeling index in breast cancer. *Int. J. Cancer*, **33**, 441–445.

WALKER, R.A. & CAMPLEJOHN, R.S. (1986). DNA flow cytometry of human breast carcinomas and its relationship to transferrin and epidermal growth factor receptors. *J. Pathol.*, **150**, 37–42.

WATERFIELD, M.D., MAYERS, E.L.V., STROOBANT, P., BENNET, P.L.P., YOUNG, S., GOODFELLOW, P.N., BANTING, G.S. & OZANNE, B. (1982). A monoclonal antibody to the human epidermal growth factor receptor. *J. Cell Biochem.*, **20**, 149–161.

WRIGHT, C., ANGUS, B., NICHOLSON, S., SAINSBURY, J.R.C., CAIRNS, J., GULLICK, W.J., KELLY, P., HARRIS, A.L. & HORNE, C.H.W. (1989). Expression of c-erbB-2 oncoprotein: A prognostic indicator in human breast cancer. *Cancer Res.*, **49**, 2087–2090.